# Predicting risk for nocturnal hypoglycemia after physical activity in children with type 1 diabetes

**DOI:** 10.3389/fmed.2024.1439218

**Published:** 2024-10-25

**Authors:** Heike Leutheuser, Marc Bartholet, Alexander Marx, Marc Pfister, Marie-Anne Burckhardt, Sara Bachmann, Julia E. Vogt

**Affiliations:** ^1^Department of Computer Science, ETH Zurich, Zürich, Switzerland; ^2^Machine Learning and Data Analytics (MaD) Lab, Department Artificial Intelligence in Biomedical Engineering, Friedrich-Alexander-Universität Erlangen-Nürnberg (FAU), Erlangen, Germany; ^3^Department of Biosystems Science and Engineering, ETH Zurich, Basel, Switzerland; ^4^Department of Statistics, Research Center Trustworthy Data Science and Security of the University Alliance Ruhr, TU Dortmund University, Dortmund, Germany; ^5^Pediatric Pharmacology and Pharmacometrics, University Children's Hospital Basel, Basel, Switzerland; ^6^Department of Clinical Research, University of Basel, Basel, Switzerland; ^7^Pediatric Endocrinology and Diabetology, University Children's Hospital Basel, Basel, Switzerland; ^8^SIB Swiss Institute of Bioinformatics, Ecublens, Switzerland

**Keywords:** diabetes management, digital health, machine learning, supervised learning, biomedical signal processing

## Abstract

Children with type 1 diabetes (T1D) frequently have nocturnal hypoglycemia, daytime physical activity being the most important risk factor. The risk for late post-exercise hypoglycemia depends on various factors and is difficult to anticipate. The availability of continuous glucose monitoring (CGM) enabled the development of various machine learning approaches for nocturnal hypoglycemia prediction for different prediction horizons. Studies focusing on nocturnal hypoglycemia prediction in children are scarce, and none, to the best knowledge of the authors, investigate the effect of previous physical activity. The primary objective of this work was to assess the risk of hypoglycemia throughout the night (prediction horizon 9 h) associated with physical activity in children with T1D using data from a structured setting. Continuous glucose and physiological data from a sports day camp for children with T1D were input for logistic regression, random forest, and deep neural network models. Results were evaluated using the F2 score, adding more weight to misclassifications as false negatives. Data of 13 children (4 female, mean age 11.3 years) were analyzed. Nocturnal hypoglycemia occurred in 18 of a total included 66 nights. Random forest using only glucose data achieved a sensitivity of 71.1% and a specificity of 75.8% for nocturnal hypoglycemia prediction. Predicting the risk of nocturnal hypoglycemia for the upcoming night at bedtime is clinically highly relevant, as it allows appropriate actions to be taken—to lighten the burden for children with T1D and their families.

## 1 Introduction

Type 1 Diabetes (T1D) affects more than 8 million people worldwide, 1.5 million of them being younger than 20 years of age (1, 2). The disease results from autoimmune destruction of pancreatic beta cells leading to insulin deficiency. The missing hormone insulin needs to be replaced several times a day, either with subcutaneous injections or an insulin pump. Good blood glucose control is important to avoid acute and long-term complications (3). However, especially for young children and adolescents, this represents an everyday challenge (4). Low blood sugar (hypoglycemia) is the most feared and common acute complication of T1D (5, 6), and the constant risk of hypoglycemia represents a great burden, in particular for children and their caregivers (7).

Bachmann et al. (8) showed that asymptomatic nocturnal hypoglycemia is frequent, and episodes often are prolonged for several hours. The most important risk factor for hypoglycemia during the night was physical activity during the day. The risk of nocturnal hypoglycemia increased with vigorous-intensity physical activity (8, 9). On the other hand, regular physical activity is essential in long-term diabetes treatment (10) and has many additional beneficial effects (11). Simultaneously, people with diabetes need to know how to avoid exercise-induced hypoglycemia, as physical activity has a direct and delayed glucose-lowering effect (12). Physical activity induces different glucose responses in different individuals and depends on characteristics of the activity itself, like type, duration, and intensity (10). Therefore, with the current state of knowledge, it is challenging to provide the right personalized recommendations to prevent exercise-associated hypoglycemia, in particular, late onset post-exercise hypoglycemia. Developing preventive measures to avoid such nocturnal hypoglycemia can increase the children's safety overnight, improve sleep quality, and quality of life of the children and caregivers.

Several machine learning approaches exist in the literature to determine hypoglycemia—the review of (13) summarizes the current state-of-the-art, considering in total 79 studies. 31 of them focused on T1D. The most used machine learning method for hypoglycemia prediction was logistic regression (in 28 studies), followed by random forest (in 14 studies). Further applied algorithms incorporated support vector machines (SVMs), autoregressive and neural networks, and XGBoost. About 50% of the considered studies used continuous glucose monitoring (CGM) data or CGM-derived parameters for hypoglycemia prediction. Of these studies, twelve included parameters of physiological signals, and seven included parameters of exercise and physical activity. Previous work focused on different prediction horizons for hypoglycemia, such as short-term (< 180 min), mid-term (180 min to 24 h), and long-term (several days, months, or even years) (13). In the area of nocturnal hypoglycemia prediction, prediction horizons between 15 min and 7 h or nighttime without an exact length or start and end time were considered: 15 min (14–16), 30 min (14–17), 45 min (15, 16), 1 h (15, 16, 18), 3 h (19), 6 h (19–26), 7 h (27), or nighttime without an exact length or start and end time (28).

Studies focusing on nocturnal hypoglycemia prediction in children and adolescents are scarce (16, 18, 22, 23). Sampath et al. (22) and Tkachenko et al. (23) used the ChildrenData dataset that contains data from about 179 children, however, no CGM data were available. The available *n* = 476 records contained nine blood glucose measurements of the following distinct time points within 24 h: 08:00, 11:30, 13:30, 16:00, 18:00, 21:00, 00:00, 03:00, and 06:00. The three measurements at 00:00, 03:00, and 06:00 were used to identify if nocturnal hypoglycemia was present, while the other measurements of the day formed the input for the nocturnal hypoglycemia prediction. Both used a method consisting of aggregating ranking algorithms with a stochastic model. Dave et al. (16) and Duckworth et al. (18) used data with CGM for a prediction horizon of up to 60 min. The dataset used by (16) consisted of 112 participants (aged 1–21 years). They used logistic regression and random forest on data collected over 90 days. The dataset used by (18) contained 153 participants (aged 14–24 years). They obtained best results with the XGBoost framework that uses an ensemble of weak learners which are trained stagewise through gradient boosting.

In this study, the primary objective was to predict nocturnal hypoglycemia associated with physical activity in children with T1D using CGM and physiological data acquired during day and night in a structured setting. We incorporate the children's particularities like longer sleep duration, focusing on the entire night (prediction horizon of 9 hours), or investigating the effect of previous physical activity. Second, as the children performed various structured physical activities during the day in the dataset that we are considering, we want to analyze if including data from a wearable device improves the outcomes. Third, as more advanced machine learning techniques such as deep learning are currently underrepresented in literature, we want to investigate the performances of Deep Neural Network (DNN) models like Recurrent Neural Networks (RNNs) and Multilayer Perceptron (MLP) compared to the most used approaches in literature like logistic regression and random forest.

## 2 Materials and methods

**Figure 1:**
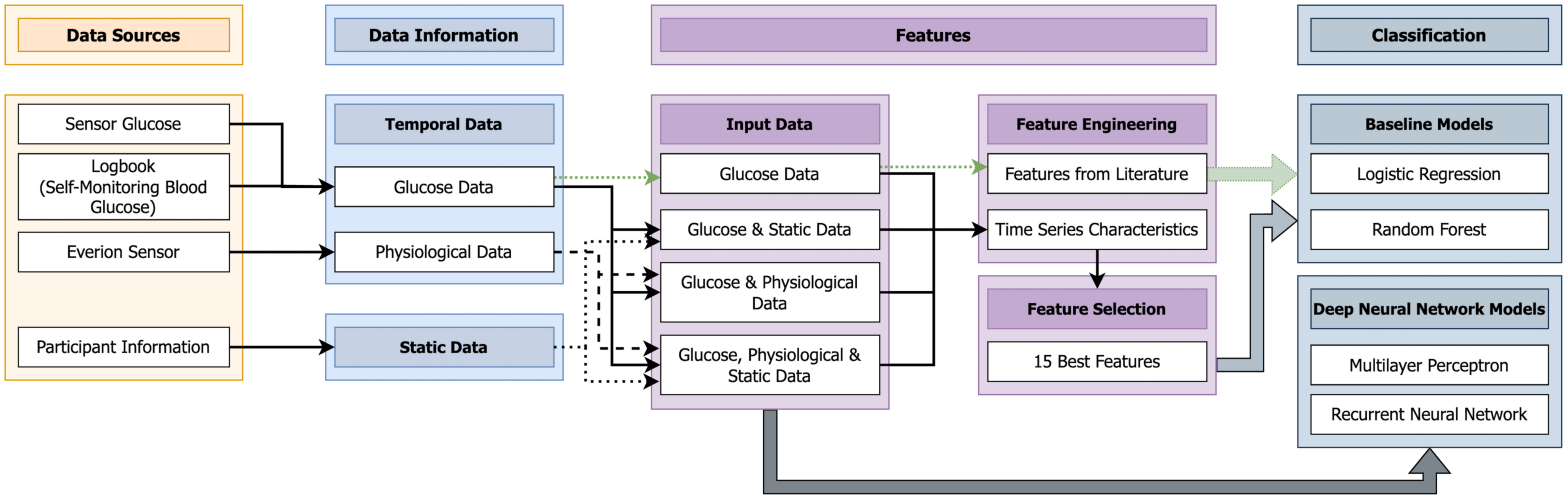
Machine learning pipeline implemented to predict nocturnal hypoglycemia (10 pm to 7 am) using data from the day (10 am–10 pm).

This part describes the used data and presents the implemented machine learning pipeline (Figure 1).

### 2.1 Data

Data of children with T1D participating in a one-week sports day camp were considered (29). The responsible Ethics Committee [Ethikkommission Nordwest- und Zentralschweiz (EKNZ)], Gesuchsnummer: 2020 - 00543, approved the study. For the data acquisition, the hardware equipment contained of a glucose sensor [intermittently scanned continuous glucose monitoring (isCGM), Freestyle libre 2 (Abbott Diabetes Care Inc., Alameda, US) or a CGM device, Dexcom (Dexcom, San Diego, US)] where the sensor was put into the subcutaneous tissue on the abdomen or the upper arm. The CGM device saved data every 5 min, and the isCGM device every 15 min. With all glucose sensors used, intersitital glucose was measured continuously. With the intermittent scanning system, however, the values are only displayed on demand, while they are visible constantly with the CGM systems. The accuracy of the sensors is comparable with mean absolute relative difference (MARD) values between 9.0% (Dexcom) and 9.7% (Freestyle libre 2) (30–34). The values are therefore reliable and comparable, even if different devices were used. Also, we trusted the devices. The alarms were not used during the day because hypoglycemia was well perceived by the participants and glucose checks were performed regularly.

In addition to CGM devices, the children were equipped with a physiological wearable sensor (Everion, Biofourmis, Boston, US). This sensor is a CE-certified research device and captured 22 signals and seven associated quality measures in real-time. In this work, we selected ten signals—and their associated quality measures, if available—for further processing (marked in **bold** in Table 1). The sampling rate was 1 Hz. Two sensors were used for each study participant and worn alternately. At the beginning of each study day (around 9 am), the Everion sensors were changed. The Everion sensor was attached to the upper arm (right or left) with an appropriately sized armband.

**Table 1:** The obtained signals when measuring with the physiological wearable sensor (Everion).

**Measured signal**
Activity classification*
Activity score
Barometer pressure
**Blood pulse wave**
**Core temperature** ^*^
Energy*
**Galvanic Skin Response (GSR) electrode**
Health score
**Heart rate** ^*^
Heart rate variability*
**Motion activity**
**Number of steps**
Oxygen saturation^*^
**Perfusion index**
Relax stress intensity score
Respiration rate*
Richness score
Sleep quality index score
Temperature barometer
Temperature local
Temperature object
Training effect score

The signals marked in bold were considered in this work. The ^*^ next to the signals states if an associated quality measure exists.

The glucose sensor and the Everion sensor were worn day and night, and the Everion sensor had to be taken off for showering and water-based activities. Additionally to sensor glucose measurements, self-monitoring blood glucose (SMBG) were performed hourly during exercise sessions, in each case of symptoms of hypoglycemia, and in case of sensor glucose values below 70 mg/dl or above 270 mg/dl. Self-monitoring of blood glucose was performed hourly during exercise i.e. at the beginning of each exercise session as recommended in the exercise management guidelines (35). As it was mandatory to perform a capillary blood glucose measurement in case of symptoms of hypoglycemia or a sensor glucose value below 70 mg/dl, a proportion of children had to do a fingerprick testing. It turned out to be more convenient/practical to do so for all participants.

**Figure 2:**
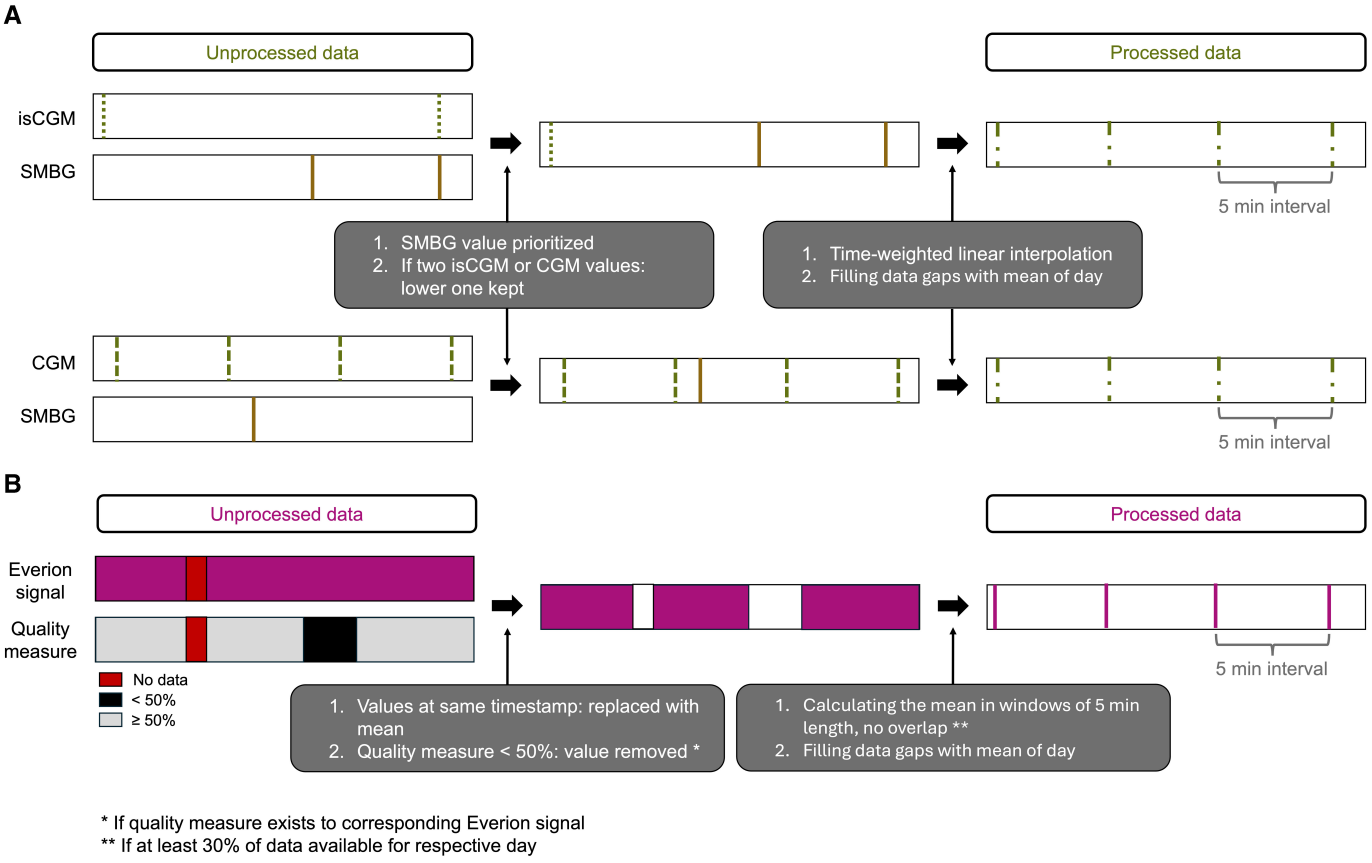
Illustration of the preprocessing steps for glucose data **(A)** and Everion data **(B)**.

Preprocessing was necessary for combining the glucose sensor data with the SMBG from the logbook and for the signals of the Everion sensor. The preprocessing steps are illustrated in Figure 2. In case of two different glucose values at the same timestamp, sensor data was overwritten with SMBG and the lower glucose sensor data were kept (Figure 2A). We decided to keep the lower value as hypoglycemia detection was the focus of this work. We curated all time series such that they have a sampling frequency of 5 min, where we use time-weighted linear interpolation to assign the glucose measurements to a time stamp. All data gaps in the glucose were filled with the respective mean of the corresponding data of the day. For data from the Everion sensor, we replaced values of duplicated timestamps with their mean (Figure 2B). For signals with an associated quality measure, we ignored values when the quality measure was less than 50% as this percentage was recommended by the manufacturer (36). Further, we considered only days with at least 30% of available data. To obtain uniform temporal data, we set the sampling interval to 5 min (corresponding to a sampling frequency of 3.33 mHz) by calculating the mean in windows of 5 min length, with no overlap of the individual windows. Similar to the glucose data, all data gaps were filled with the respective mean of the corresponding data of the day.

This above described procedure led to the exclusion of a child as not enough Everion data were measured. These processing steps resulted in using data from 13 children with a total of 66 days (ranging from 9 to 12 children per day) in this work. Of them eight children used an isCGM device and five a CGM device.

The activity classes of the parameter activity classification (Table 1) were transferred to Metabolic Equivalent of Task (MET) values (Table 2) (37) with 1 MET = 1 kcal / kg / h.

**Table 2:** The signal “Activity classification” of the Everion sensor consisted of 27 distinct activities.

**Activity**	**Assigned MET values**
Undefined	NaN
Resting	1.5
Walking flat	3
Running flat	7
Biking flat	7
Walking up	5.5
Running up	13
Biking up	10
Rowing	6
Other	NaN
Biking	7
Running	7
Walking	3
Walking down	3.5
Running down	6
Biking down	3
Sitting	1.5
Standing	2
Driving car	2.5
Driving public	1.5
Sleeping	1
Awake	1.5
Ctrl rest med ee	NaN
Measured and relevant improvement in Relax	NaN
Measured and relevant improvement in Sleep	NaN
Measured and relevant improvement in Exercise	NaN
Measured and relevant improvement in Move	NaN

We assigned, according to the “compendium of physical activities” (37) the following Metabolic Equivalent of Task (MET) values to single activities with 1 MET = 1 kcal / kg / h.

### 2.2 Machine learning algorithm

The general idea of this work was to develop a classification system to answer the research question whether nocturnal hypoglycemia can be predicted with physiological and glucose data collected during the day. The night was defined between 10 pm and 7 am. The day was defined between 10 am and 10 pm.

A hypoglycemic event was defined as either (1) a single or multiple SMBG < 3.9 mmol/l or (2) an interval greater than 15 min, in which all continuous glucose measurements were < 3.9 mmol/l (14, 16, 38). In the present data, 48 nights were found without nocturnal hypoglycemia and 18 with nocturnal hypoglycemia.

Figure 1 gives an overview of the different steps of the designed machine learning system. In this work, we use a supervised learning approach. In the training phase of a supervised machine learning system, the information about the actual label is known to the classifier. Then in the testing phase, the actual labels are predicted. The standard pipeline of a supervised machine learning model consists of the following four steps: (1) data, (2) preprocessing, (3) features, and (4) classification (39). These steps were also used in this work (Figure 1). For the last step, the classification, we decided to use two baseline classifiers, logistic regression and random forest, as these are the most used algorithms in literature for hypoglycemia prediction (13). In case of the DNN models, the third steps (features) was left out as the complete data were directly used.

**Figure 3:**
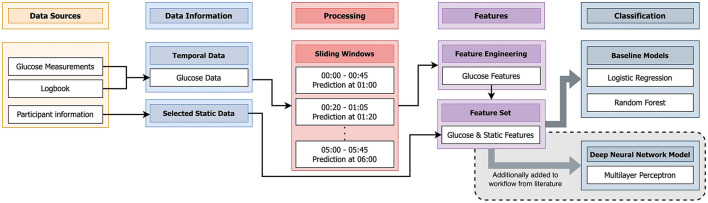
Schematic visualization of the algorithm from literature (14) that we applied to the data of this study. We additionally implemented a Deep Neural Network (DNN) [Multilayer Perceptron (MLP)] not mentioned in literature.

We used glucose measurements, the logbook, the Everion sensor, and participant information as data sources (Section 2.1). The sensor glucose measurements and the logbook data (SMBG) were combined to form the glucose data (details also available in Figure 3). The chosen signals of the Everion sensor (Table 1) form the physiological data. From the available participant information, the age, weight, height, Body mass index (BMI), and gender (male or female) were extracted to form the static data.

With the available temporal and static data, four dataset combinations were chosen as input data for the algorithms:(1) glucose data only, (2) glucose and static data, (3) glucose and physiological data and (4) glucose, physiological, and static data.

In the case of the DNN models, these four input datasets were directly used in the following two scenarios:

1. A RNN for the temporal data.
  The RNN included a masking layer followed by a bidirectional Gated Recurrent Unit (GRU) (40) layer, a dropout layer, a Long Short-Term Memory (LSTM) (41) layer, and another dropout layer.
2. A RNN for the temporal data, and a MLP for the static data.
  Both were combined afterward. The output of both the MLP and the RNN were concatenated and processed by an additional MLP. The RNN included a single LSTM layer.

Considering every distinct combination of input datasets, four different architectures were designed by composing individual network blocks. The RNN is predominantly used for sequential and temporal data, whereas the MLP finds applications in all domains of machine learning (42). Here, we can only pass data according to a predefined time-window into the MLP, which can not consider data outside of the window. RNNs on the other hand, continuously update a hidden state while processing the data. Therefore, the hidden state can potentially capture long-ranging dependencies.

To find the best architectures, considering each composition individually, the neural networks were subject to hyperparameter optimization using the Hyperband algorithm, introduced by Li et al. (43). The grid of hyperparameters to be optimized included the number of layers, number of nodes per layer, application of GRU or LSTM units, addition of bidirectionality to the recurrent units, addition of dropout layers, and masking zero-values. In order to properly treat the missing values of the input data, which have been substituted with the parameter-specific mean values, the masking ignores the mean values. Since all parameters have been standardized, the mean values are equal to zero. We introduced class weights to the loss function during training to account for the class imbalance (18 nights with nocturnal hypoglycemia, 48 nights without nocturnal hypoglycemia). We chose the class weights inversely proportional to their respective frequencies. This led to increased attention to the underrepresented class samples and forced the model to improve equally in both classes during the training phase. We used the Rectified Linear Unit (ReLU) activation function throughout the dense layers to avoid computational complexity and vanishing gradients, as suggested in literature (44, 45). The hyperbolic tangent (tanh) activation function was used for the recurrent layers based on research conducted by Chung et al. (46) and Hochreiter and Schmidhuber (41), as it has the property of limiting the issue of exploding gradients while still providing a strong gradient. We used adaptive moment estimation (Adam) as the optimizer with a learning rate of 0.001 since the optimizer Adam performs on average better on deep learning task than than Stochastic Gradient Descent (SGD) (47, 48). We set the batch size to 1 due to the small number of samples in the dataset and the intention to counteract for overfitting and poor generalization (49). Although, Adam with batch size 1 reduces to an update rule similar to the vanilla SGD algorithm, it still preserves the features regarding the moments of the gradient (47).

In the case of the baseline classifiers, features were engineered from the four input datasets and selected before being the input to either logistic regression or random forest. Logistic regression uses a logistic function (the sigmoid function) to model the probability that a given input belongs to a particular class (50). Random forest is an ensemble learning method that operates by constructing multiple decision trees and using the majority vote of these trees to determine the correct class (50).

For the glucose data, we calculated eight features from literature (14) to reflect glucose dynamics. These were coefficient of variation, lability index, low blood glucose index, 1 h continuous overlapping net glycemic action, minimal value, the difference between the last two values, acceleration over the last values, and linear trend coefficient. In addition, we used for all four input datasets the Python library *tsfresh* to calculate time series characteristics, which was followed by a feature selection step. This library was specifically developed to create new features aggregated on temporal dependencies. With *tsfresh*, approximately 800 new features for every single initial feature were engineered. To reduce the number of features to the best 15 features, we conducted a performance-based, sequential feature selection using the Python library *scikit-learn*. The options of *scikit-learn* were set in accordance to the classifier that was used afterward.

This resulted in five different feature-data-combinations that were used for the following two baseline classifiers:

1. Logistic regression with Least Absolute Shrinkage and Selection Operator (LASSO) regularization
2. Random forest with 10 trees.

We chose to focus on these two classifiers because they are the most commonly used algorithms in the literature for hypoglycemia prediction (13), and are also applied in the field of nocturnal hypoglycemia prediction (14, 16).

### 2.3 Comparison to algorithm in literature

**Figure 4:**
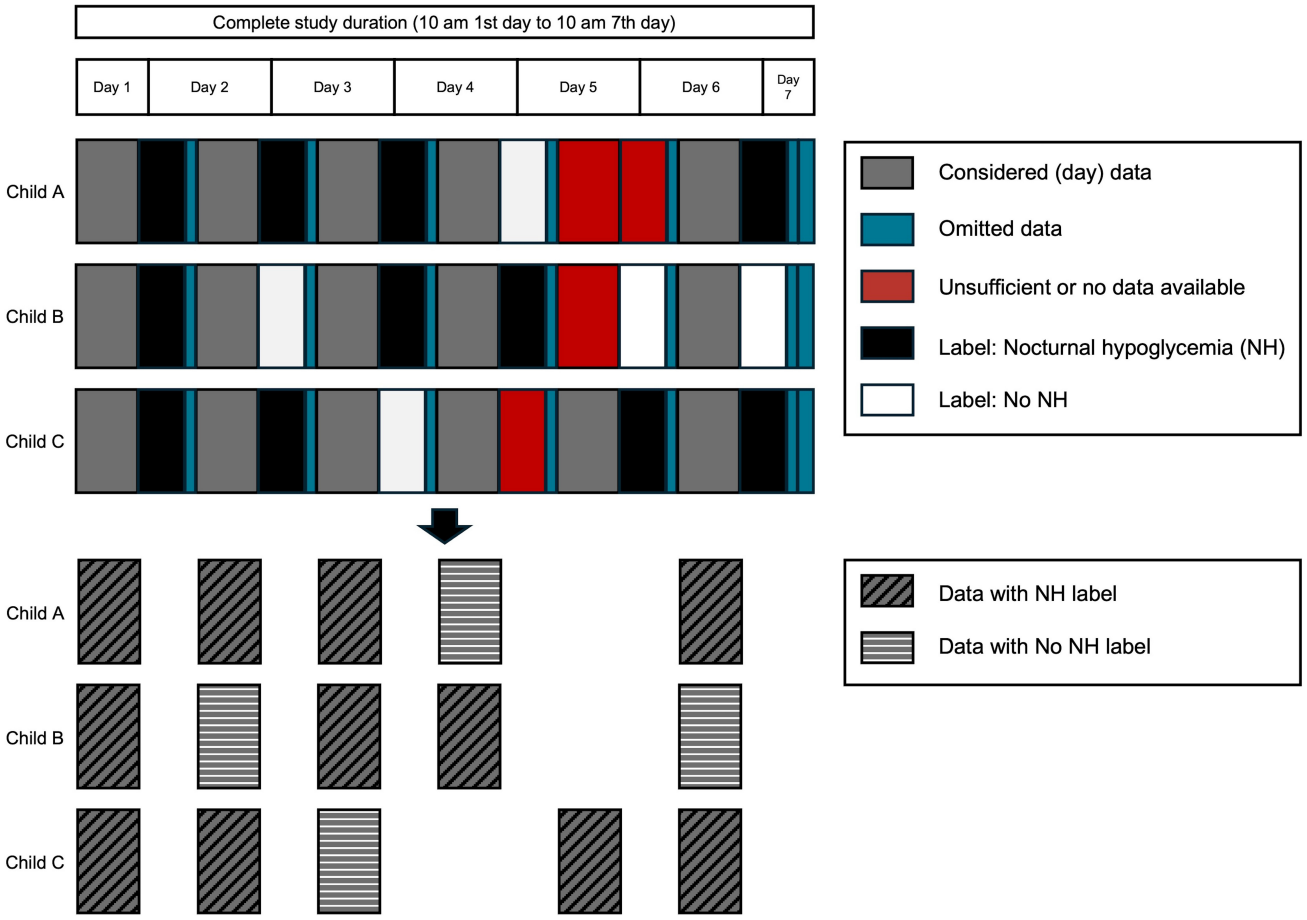
Illustration of preparing the data for the evaluation using fictitious data of three children. For each subject individually, the data is divided into distinct days (10 am to 10 pm) and nights (10 pm to 7 am). Data of the nights is used to determine if a hypoglycemic event occurred. In the ML algorithm, data of the day with the corresponding label were used.

To assess the quality of our dataset, we decided to re-implemented an algorithm in literature (14). Berikov et al. (14) applied the two most used algorithms for hypoglycemia prediction (logistic regression and random forest) in the field of nocturnal hypoglycemia and obtained best results with a prediction horizon of 15 min, which was much shorter than the used prediction horizon of 9 h in this work. Still, we considered it valuable to evaluate the quality of our dataset. We reimplemented the algorithm as described in the associated manuscript (14), adapted where necessary, and applied it to the described data of this work. Additionally, we used an adapted version using elements of the published algorithm and the algorihm of this work. More details about the reimplemented algorithm and the adapted version is given in the next paragraph. Figure 4 illustrates the steps of the algorithm from (14) applied in this work. The glucose measurements, the logbook, and participant information were included as data sources.

As described previously, the glucose data consisted of a combination of the glucose measurements and the SMBG entries of the logbook. From the participant information, age, BMI, and gender formed the selected static data. The glucose data was processed in sliding windows of the acquired nights, in the time frame of 0 am (midnight) to 6 am. Each sliding window had a duration of 45 min. The next window started 20 min after the start of the previous window. Single windows were used to predict a hypoglycemic event exactly 15 min after the end of the window. In each window for the glucose data, we extracted the same eight features from literature (14) as mentioned in Section 2.2. These features were combined with the selected static data to form the feature set. Similar to (14), a logistic regression with LASSO regularization and random forest were trained with this feature set. Additionally, not mentioned by (14), we decided to train a MLP with the same feature set.

### 2.4 Performance measures and evaluation

We compared all predicted nocturnal hypoglycemic events with all true nocturnal hypoglycemic events (Table 3).

**Table 3:** Confusion matrix for the two classes *Nocturnal Hypoglycemia* (NH) and *No Nocturnal Hypoglycemia* (no NH).

		**Predicted**	
		**no NH**	**NH**
**True**	**no NH**	True Negatives (TN) (correct rejection)	False Positives (FP) (overestimation)
	**NH**	False Negatives (FN) (underestimation)	True Positives (TP) (hit)

The classification systems were compared regarding six evaluation metrics: the specificity, the sensitivity, the precision, the F2 score, the F1 score, and the Area Under the Receiver Operating Characteristic Curve (AUC) score (Table 4). The specificity represents the proportion of nights without nocturnal hypoglycemia, which were correctly classified as nights without nocturnal hypoglycemia. The sensitivity (also known as recall) gives the proportion of nights with nocturnal hypoglycemia, which were correctly classified as nights with nocturnal hypoglycemia. The precision (also known as positive predictivity) gives the proportion of correctly classified nights with nocturnal hypoglycemia over all nights predicted as nights with nocturnal hypoglycemia.

**Table 4:** Definition of metrics for assessing the performance of the implemented classifiers.

**Metric**	**Definition**
**Specificity**	** $\frac{\text{TN}}{\text{TN}+\text{FP}}$ **
**Sensitivity**	$\frac{\text{TP}}{\text{TP}+\text{FN}}$
**Precision**	$\frac{\text{TP}}{\text{TP}+\text{FP}}$
**F**_**β**_ **score**	$\left(1+\beta^{2}\right)\cdot \frac{\text{Precision}\cdot \text{Sensitivity}}{\left(\beta^{2}\cdot \text{Precision}\right)+\text{Sensitivity}}$, with either β = 2 (F2 score) or β = 1 (F1 score)
**AUC score**	Area Under the Receiver Operating Characteristic Curve

TN, true negatives; FP, false positives; FN, false negatives; TP, true positives.

The F1 score gives precision and sensitivity the same weight, is broadly used in literature, and is known to be a good metric for imbalanced classification tasks (51, 52). The F_β_ score is a generalization of the F1 score adding the configuration parameter β. In the F2 score, β is set to 2, giving more weight to sensitivity than to precision (52). In the present case and generally in medical settings, the misclassification as false negative (underestimation) is worse than the misclassification as false positive (overestimation). We decided to use the F2 score as the metric on the validation set when it came to stopping the training or in determining the relevant parameteres and features. The AUC score gives the area under the receiver operating characteristic curve.

**Figure 5:**
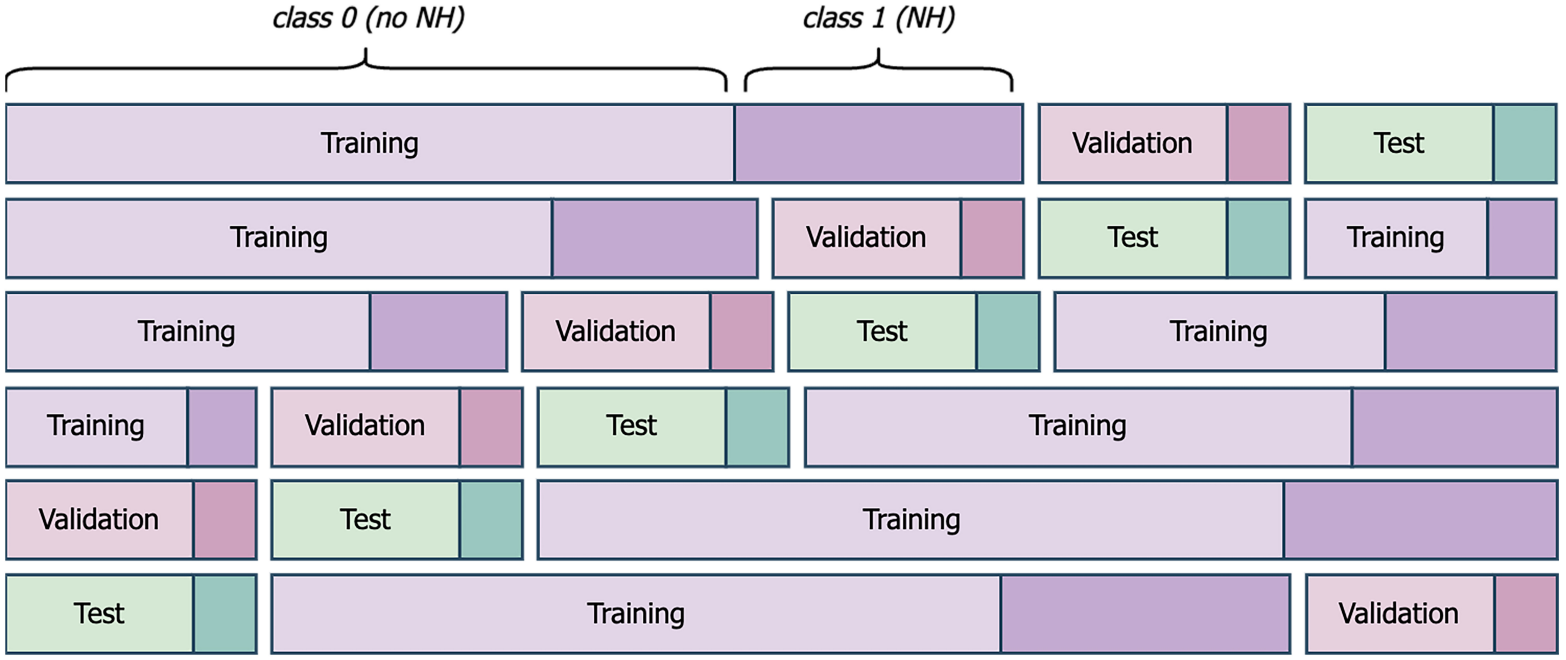
Schematic presentation of six-fold cross-evaluation for one of the five executions. Exemplary folds and splits of the dataset into training, validation, and test set are given. Class 0 comprises all nights without nocturnal hypoglycemia (NH). Class 1 represents all nights with NH.

**Figure 6:**
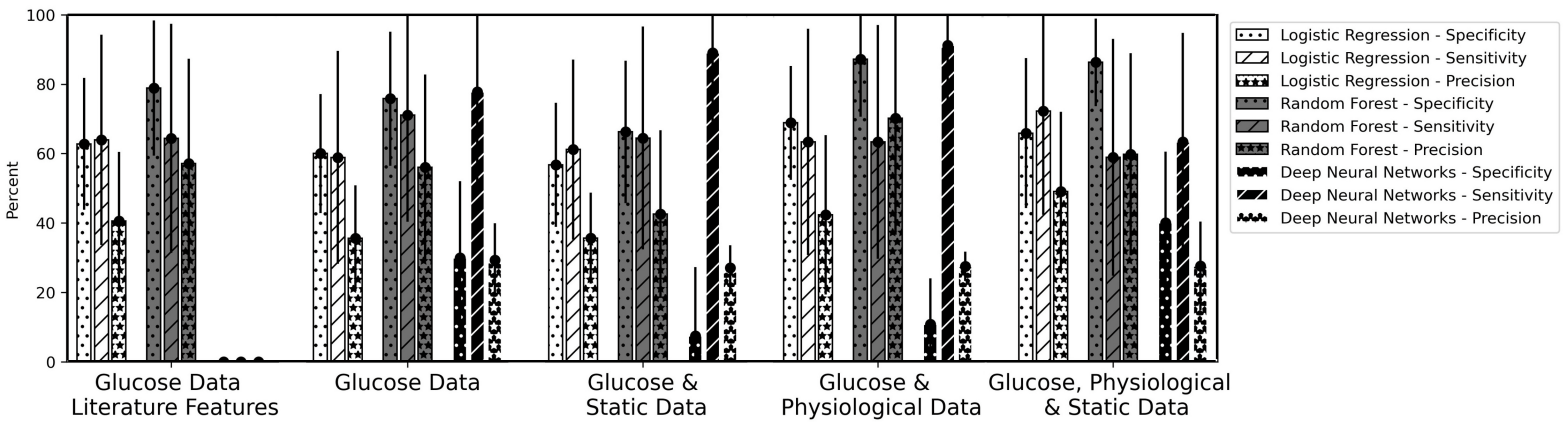
Mean values of Specificity, Sensitivity, and Precision for the overnight prediction (prediction horizon: 10 pm to 7 am) given for logistic regression, random forest and the deep neural network models. The bars are calculated as average values of the five runs for the six-fold cross-validation. The error bars belong to the mean of the standard deviation of the five runs.

Each machine learning model was subjected to six-fold cross-evaluation to counteract the small sample size and to support reliable and reproducible results. The available data after preprocessing (Figure 3) was divided into individual days with their corresponding label from the following night (Figure 5). 66 days with available label from the night formed the dataset. The dataset was shuffled and divided into six-folds, with five folds forming the training set and one fold forming the test set. In each case, 20% of the training set was subtracted to form the validation set for the DNN models (Figure 6). We considered a population-based approach and it was possible, that data from the same child was in the training, validation and test set. The shuffle and split were done in a stratified fashion so that classes were distributed almost identically among the different sets. This above mentioned procedure was executed five times, using the same randomly generated partitions of the data for all dataset combinations. The results are given averaged over all executions with the belonging standard deviation.

## 3 Results

Table 5 gives the characteristics of the 13 children that were included in the data analysis. Table 6 shows the mean values with standard deviation of three performance measures (Table 4) for the developed machine learning pipeline (Figure 1). Figure 6 displays the three remaining performance measures, the specificity, the sensitivity, and the precision, to the same machine learning pipeline as in Table 6. Table 6 gives the obtained results for the (adapted) implemented algorithm from literature (14) (Figure 4).

**Table 5:** Participant characteristics for all *N* = 13 children (4/13≃30.7% female).

	**Mean ± SD**	**Range**
Age (years)	11.3 ± 2.2	[7.5, 13.9]
BMI (kg/m^2^)	19.8 ± 4.4	[13.2, 27.7]
Diabetes duration (years)	4.1 ± 2.8	[0.9, 9.5]
HbA1c (%)	7.1 ± 0.9	[5.1, 8.5]

Diabetes duration is the time since Type 1 Diabetes (T1D) diagnosis. HbA1c refers to glycated hemoglobin measured in the range of 54 days before the first study day and 74 days after the first study day. SD, Standard deviation; BMI, Body mass index.

**Table 6:** Mean values ± standard deviation (in percent) of the performance measures (F2 and F1 scores, and AUC) for the overnight prediction (10 pm to 7 am) given for the baseline models logistic regression (LR) and random forest (RF) and the Deep Neural Network (DNN) models.

			**Time series characteristics**			
**Data sets**		**Glucose literature features**	**Glucose**	**Glucose & static**	**Glucose & physiological**	**Glucose, physiological & static**
LR	F2	**56.4** **±** **20.9**	50.6 ± 24.0	52.1 ± 19.8	56.3 ± 27.8	**63.0** **±** **23.7**
	F1	56.0 ± 12.0	42.7 ± 18.6	43.6 ± 15.4	49.3 ± 24.2	54.8 ± 20.3
	AUC	68.3 ± 13.1	59.4 ± 15.4	58.9 ± 14.0	66.0 ± 17.2	69.0 ± 16.2
RF	F2	40.8 ± 23.1	**64.4** **±** **26.0**	56.0 ± 26.3	61.3 ± 30.3	56.6 ± 29.6
	F1	47.3 ± 25.2	58.3 ± 22.6	48.3 ± 22.3	60.9 ± 27.6	55.4 ± 25.9
	AUC	66.4 ± 11.9	73.5 ± 14.5	65.3 ± 15.3	75.2 ± 16.6	72.6 ± 14.9
DNN	F2	n.a.	57.5 ± 19.1	**59.5** **±** **12.9**	**62.0** **±** **11.3**	49.1 ± 22.3
	F1	n.a.	41.9 ± 14.1	40.4 ± 7.5	42.1 ± 6.9	37.6 ± 16.6
	AUC	n.a.	53.9 ± 13.7	48.2 ± 8.6	51.0 ± 7.6	51.7 ± 14.0

The standard deviations are calculated as average values of the five runs. For LR and RF, different features were calculated (Literature Features or Time Series Characteristics). The used machine learning pipeline is illustrated in Figure 1. AUC, Area Under the Receiver Operating Characteristic Curve; n.a., not applicable. Best F2 scores per data set are marked in bold.

In the implemented machine learning pipeline, different feature-data-combinations were the input for the classification task using logistic regression, random forest or DNN. Best results (F2 score 64.4%) were obtained with time series characteristics of glucose data using random forest (Table 6). Time series characteristics of glucose, physiological, and static data with logistic regression (F2 score 63.0%) achieved a similar F2 score then the DNN with glucose and physiological data (F2 score 62.0%) (Table 6). Single high values (above 80%) were also reached for specificity and sensitivity for a variety of options (Figure 6).

## 4 Discussion

The focus of this work was the prediction of nocturnal hypoglycemia (prediction horizon 9 h) concentrating on children with T1D. We used CGM, physiological data from a wearable device, and children characteristics.

### 4.1 General discussion

Despite achieving single high values (above 80%) for specificity and sensitivity for a variety of options (Figure 6), the selected and implemented machine learning pipeline could not achieve values over 65% for the combined F2 score, e.g. due to the small data set. The high standard deviations for all models and dataset combinations indicate that, depending on the distribution of the dataset (cross-validation), it is unclear which model is actually the best (Table 6, error bars in Figure 6).

Comparing the baseline models to DNNs, it is apparent that the DNNs achieved smaller standard deviations. This shows the potential of the DNN models as this indicates the more consistent performance over all six-folds for the five runs. The consistency suggests that DNNs better capture complex patterns and relationships than traditional models. DNN models typically require large datasets—ideally large in number of samples to generalize well in tuning the parameters and in the number of children, study days, and class balance to learn a comprehensive representation. We assume that either (1) combining different datasets into a larger one or (2) applying transfer learning would improve the results for the DNN models. In transfer learning, either this dataset is used to train the model, and fine-tuning is done on the other dataset, or vice versa.

Investigating the results for incorporating physiological data, it is visible that there is an improvement in the F2 scores for the baseline models logistic regression and random forest (Table 6). As mentioned in the last paragraph, we assume that DNN models need a larger dataset to realize their full potential. An improvement was not (random forest) or, at most, slightly (logistic regression) visible when only static data was included. Our assumption here is that static data describes the children's general characteristics and could impact the general risk for hypoglycemia. In contrast, it does not improve the short-term prediction for the upcoming night. When assessing the upcoming night, the influence of physiological data is greater, particularly regarding activity and energy expenditure during the day, and has a direct impact on blood glucose at night, which is also reflected in the general trend.

Improvements for future work could be targeted hand-crafted features, with specific feature selection, and incorporating ML strategies to enlarge the dataset.

### 4.2 Comparison to reimplemented algorithm from literature

For evaluating the quality of our dataset, we reimplemented an algorithm from literature (14) that uses logistic regression and random forest for a prediction horizon of 15 min (Figure 4). berikov et al. (14) achieved best results with random forest (Specificity 91.4%, Sensitivity 94.5%, AUC score 0.97). Best results using our dataset were attained with logistic regression (Specificity 90.4%, Sensitivity 90.4%, F2 score 63.9%, AUC score 90.7%) (Table 7).

**Table 7:** Mean values of the performance measures for the 15 min prediction during the night (only data considered midnight to 6 am).

	**Logistic regression**	**Random forest**	**Multilayer perceptron**
Specificity	90.4%	**98.9%**	77.1%
Sensitivity	**90.4%**	20.5%	61.1%
Precision	29.2%	45.0%	**50.0**%
F2 score	**63.9**%	23.0%	58.5%
F1 score	43.3%	35.8%	**55.0%**
AUC score	**90.7%**	59.7%	69.1%

The models logistic regression and the random forest are exact re-implementations of (14). The Multilayer Perceptron (MLP) as Deep Neural Network (DNN) model was additionally implemented. The used procedure is illustrated in Figure 3. Best performance measures per ML model are marked in bold.

Single values for specificity, sensitivity, and precision range from 20.5% to 98.9%, for the F2 score from 23.0% to 63.9%. The previously best performing algorithm random forest achieves the worst results. Differences between the reimplemented algorithm and the other algorithm presented in this work are 1) different prediction horizon, 2) selected hand-crafted features are input to random forest and logistic regression, and 3) different data were used. Data between midnight to 6 am were used. Previously this data served only to determine one of the two classes: Nocturnal Hypoglycemia and No Nocturnal Hypoglycemia. Hence, more samples were used in the 15 min prediction. This could indicate that the effect of exercise is relevant whereas cannot be depicted with data only during the night and emphasizes that the combination—data, if needed features, and classifiers— is relevant.

Berikov et al. (14) did not present their results using precision or the F2 score. They used a dataset of 406 adults (aged 18 to 70 years) having a mean CGM duration of each participant of 6.7 days. Since the sensitivity and specificity in the different works are in the same ranges, we conclude that our dataset is comparable to the larger dataset (14) despite the smaller sample size and other differences (participants were children, mean CGM duration of 5.1 days).

### 4.3 Comparison with literature

Three studies from the literature (20, 21, 26) concentrated on nocturnal hypoglycemia prediction (prediction horizon of 6 h for all three studies) and included wearable data. Bertachi et al. (26) received best results (78.75% median sensitivity, 82.15% median specificity) with SVM, evaluating the generated classifiers with $Gmean=\sqrt{Sensitivity\cdot Specificity}$. Parcerisas et al. (20) used the same dataset as Bertachi et al. (26) and achieved with SVM a median sensitivity of 74% and a median specificity of 77% for their population models, evaluating the generated models with the F1 score. Veh´ et al.(21) used artificial neural networks and obtained a mean sensitivity of 44.0% and mean specificity of 85.9%. In this work, mean sensitivity and specificity to the best F2 score were 77.8% and 62.5%, respectively (Table 6). If we consider the differences in the studies, such as children compared to adults or longer prediction horizon, we conclude that the results in this work are comparable to or even exceed the results in the literature.

We used data collected during a sports day camp. During the day, the children were supervised by pediatric endocrinologists. The children were supervised by their parents in the evenings and at night. This study setting is less controlled than an inpatient hospitalized setting as used in literature (14, 22, 23). Other studies use an even less controlled outpatient setting, where participants continue their daily routines and come to the study center only at agreed times (16, 18, 20, 26). The chosen study setting allows the imitation of everyday daily life but offers opportunities for intervention and offers information about meals and insulin doses. Data from a less supervised setting including a higher number of participants will also be considered in the future.

Population-based models and individual-based (or personalized) models are used in the literature (20). Population-based models describe models in which one or more participants are not seen by the algorithm in the training phase. Individual-based models are models that explicitly consider data from the individual in the training phase to (ideally) produce better results for the individual. Due to the scarcity of our data, we combined all available study days and ignored the individual participant. In the future, we plan to evaluate the performance of population-based and individual-based models.

### 4.4 Limitations

In this work, we decided to use logistic regression and random forest as these were the most used algorithms in literature for hypoglycemia prediction (13). In principle, no particular classifier is appropriate for all classification tasks (No Free Lunch Theorem) (53). Hence it might be that for our research questions and the specific dataset including children a different classifier would improve the performance. For example, in future work we want to incorporate XGBoost or SVM as the algorithms have a lower complexity than DNN and have shown good performances in similar work.

We transferred the activity classes to MET values (Table 2) based on the adult compendium (37). This comprehensive list included all available activity classes. The 'Youth Compendium of Physical Activities' (54) contains only 200 activities and their assigned MET value for four different age groups (6-9, 10-12, 13-15, 16-18 years). Since not all required activity classes were available in the “Youth Compendium”, we decided to stay with the established adult compendium.

For the logistic regression and random forest, we performed a sequential forward feature selection and limited the number of features to 15. We did not investigate other feature selection approaches or perform an extensive search regarding best number of features. In future work, we will address this issue together with newly created hand-crafted physiological features.

### 4.5 Outlook

Vu et al. (19) noted, the difference between the early phases of sleep and the late phases of sleep has not been widely studied in the field of predicting nocturnal hypoglycemia. The literature often distinguishes between the first three hours of sleep (midnight to 3 am) and the remaining three hours of sleep (3 am to 6 am). During the beginning of sleep, slow wave sleep (SWS)—like non-rapid eye movement (REM) sleep—dominates, while REM sleep becomes more prevalent toward the end of sleep (55). The brain's need for glucose is significantly decreased during SWS, reaching a minimum level that is distinctively lower than during wakefulness or REM sleep (55). Research studies (55, 56) indicate that more nocturnal hypoglycemia events are present during late sleep. In addition, adults and children with T1D report worse sleep quality than healthy controls (19). In the future, we want to investigate nocturnal hypoglycemia prediction in the early phases of sleep compared to the late phases of sleep, adjusted to data from children that have a higher sleep requirement. For this, we will also consider (night) parameters of the wearable sensor like *sleep quality index score* (Table 1).

The logbook also contains information about the structured sports activities that were performed on a daily basis. The inclusion of this information in combination with the activity classes and other activity parameters could be used to establish rules for hand-crafted features for the signals of the Everion sensor. This could then lead to better results when incorporating physiological data from the wearable sensor.

Lehmann et al. (57) created a decision tree-based machine learning method to detect hypoglycemia in unknown individuals using only data collected by non-invasive wearables. They concentrated on adults with diabetes. Their pilot study indicates that wearable data can provide valuable insights into hypoglycemia prediction, even without using CGM. Analyzing signals from the Everion sensor separately from CGM data and then combining them with CGM and child characteristics could be another aspect of future work.

In addition, there are other options for using the signals of the Everion sensor (Table 1). Studies (58–60) have shown a relationship between heart rate variability and hypoglycemia. In future work, we want to investigate this association. Further, we want to incorporate insulin and carbohydrates in the future as it was shown in literature that the inclusion increased the performance (16). We want to also compare the performance of the developed algorithms on public datasets, e.g. the OhioT1DM Dataset (61) or data from the CITY study (18).

## 5 Conclusion

In this work, we utilized a dataset recorded in a structured setting to assess the risk of nocturnal hypoglycemia associated with physical activity in children with T1D. In contrast to previous studies we aimed for longer-term predictions, i.e. up to 9 h vs. up to 1 h. From our point of view, the results obtained in this study are acceptable with a sensitivity of the best F2 score close to 80%. Understanding the hypoglycemia risk for the entire upcoming “critical” night is clinically relevant as it permits children and their parents to either sleep soundly or to take appropriate action such as reducing basal insulin doses, administering additional carbohydrates, or scheduling a nocturnal glucose measurement.

## Data Availability

The presented datasets in this article are not publicly available to ensure participant privacy. Requests to access the dataset should be directed to the corresponding authors.
